# Spatiotemporal correlation enhanced real-time 4D-CBCT imaging using convolutional LSTM networks

**DOI:** 10.3389/fonc.2024.1390398

**Published:** 2024-08-05

**Authors:** Hua Zhang, Kai Chen, Xiaotong Xu, Tao You, Wenzheng Sun, Jun Dang

**Affiliations:** ^1^ School of Biomedical Engineering, Southern Medical University, Guang Zhou, Guangdong, China; ^2^ Guangdong Provincial Key Laboratory of Medical Image Processing, Southern Medical University, Guang Zhou, Guangdong, China; ^3^ School of Artificial Intelligence, Chongqing University of Technology, Chongqing, China; ^4^ Department of Radiation Oncology, The Affiliated Hospital of Jiangsu University, Zhenjiang, Jiangsu, China; ^5^ Department of Radiation Oncology, The Second Affiliated Hospital, School of Medicine, Zhejiang University, Hangzhou, Zhejiang, China; ^6^ Department of Radiation Oncology, National Cancer Center/National Clinical Research Center for Cancer/Cancer Hospital & Shenzhen Hospital, Chinese Academy of Medical Sciences and Peking Union Medical College, Shenzhen, China

**Keywords:** ConvLSTM, PCA, radiation therapy, 4D-CBCT, spatiotemporal

## Abstract

**Purpose:**

To enhance the accuracy of real-time four-dimensional cone beam CT (4D-CBCT) imaging by incorporating spatiotemporal correlation from the sequential projection image into the single projection-based 4D-CBCT estimation process.

**Methods:**

We first derived 4D deformation vector fields (DVFs) from patient 4D-CT. Principal component analysis (PCA) was then employed to extract distinctive feature labels for each DVF, focusing on the first three PCA coefficients. To simulate a wide range of respiratory motion, we expanded the motion amplitude and used random sampling to generate approximately 900 sets of PCA labels. These labels were used to produce 900 simulated 4D-DVFs, which in turn deformed the 0% phase 4D-CT to obtain 900 CBCT volumes with continuous motion amplitudes. Following this, the forward projection was performed at one angle to get all of the digital reconstructed radiographs (DRRs). These DRRs and the PCA labels were used as the training data set. To capture the spatiotemporal correlation in the projections, we propose to use the convolutional LSTM (ConvLSTM) network for PCA coefficient estimation. For network testing, when several online CBCT projections (with different motion amplitudes that cover the full respiration range) are acquired and sent into the network, the corresponding 4D-PCA coefficients will be obtained and finally lead to a full online 4D-CBCT prediction. A phantom experiment is first performed with the XCAT phantom; then, a pilot clinical evaluation is further conducted.

**Results:**

Results on the XCAT phantom and the patient data show that the proposed approach outperformed other networks in terms of visual inspection and quantitative metrics. For the XCAT phantom experiment, ConvLSTM achieves the highest quantification accuracy with MAPE(Mean Absolute Percentage Error), PSNR (Peak Signal-to-Noise Ratio), and RMSE(Root Mean Squared Error) of 0.0459, 64.6742, and 0.0011, respectively. For the patient pilot clinical experiment, ConvLSTM also achieves the best quantification accuracy with that of 0.0934, 63.7294, and 0.0019, respectively. The quantification evaluation labels that we used are 1) the Mean Absolute Error (MAE), 2) the Normalized Cross Correlation (NCC), 3)the Structural Similarity Index Measurement(SSIM), 4)the Peak Signal-to-Noise Ratio (PSNR), 5)the Root Mean Squared Error(RMSE), and 6) the Absolute Percentage Error (MAPE).

**Conclusion:**

The spatiotemporal correlation-based respiration motion modeling supplied a potential solution for accurate real-time 4D-CBCT reconstruction.

## 1 Introduction

Stereotactic radiotherapy (SBRT) is commonly used in routine clinical radiation therapy circumstances, especially for early-stage cancer such as lung cancer (1). The high dose rate of the SBRT beam also brings high risk for moving targets (e.g., lung cancer). Hence, accurate image guidance plays a crucial role in precise lung SBRT. In clinical routine, the most common image guidance tool is the integrated 3D Cone Beam CT (CBCT) imaging system (2). However, conventional static 3D-CBCT is unable to provide qualified 4D lung motion during respiration.

Four-dimensional cone beam CT (4D-CBCT) imaging has been developed to address this issue. 4D-CBCT can supply temporal image sequences for moving organs such as the lung. Conventional analytical 4D-CBCT methods, such as the McKinnon–Bates (MKB) algorithm, are widely used in commercial linear accelerators. However, the image quality suffered from reduced contrast and the inevitable motion blurring induced by the time-averaged prior image (3). Another type of 4D-CBCT reconstruction method is the image deformation-based scheme (4). For these kinds of methods, the deformation vector fields (DVFs) calculation/estimation between the 0% phase and each other phase is critical to achieve the final accurate 4D-CBCT. The DVF optimization process is quite time consuming, and it raises a blind treatment risk for initiating radiation pneumonia (5). Both the above-mentioned analytical and deformable-based 4D-CBCT reconstructions all use the full 360° range acquired projections. Recently, online real-time CBCT estimation/reconstruction via single or only a few X-ray projections has attracted more interest. It benefits oncologists not only fast but also pretty low-dose real-time 4D-CBCT images compared with the conventional full projection-based 3D-CBCT (6).

The 2D- to 4D-CBCT estimation has been previously studied by many groups in the past decades. Li (7) proposed a motion model (MM) to predict 4D-CBCT via forward matching between 3D volumes and 2D X-ray projections. You (8) reported a motion model free deformation (MM-FD) scheme to introduce free deformation alignment for promoting 4D-CBCT estimation accuracy. One limitation of these iterative approaches is that they are quite time consuming. On the other aspect, Xu (6) reported a linear model for predicting 4D-CBCT via DRR (Digital Reconstructed Radiography) and validated it with digital and physical phantom experiments. However, the proposed linear model mismatches with the complex relationship between the intensity variation and the real breathing motion. Wei (9, 10) proposed a Convolutional Neural Network (CNN)-based framework to extract the motion feature from 2D DRRs to corresponding 3D-CBCT (e.g., one phase of 4D-CBCT). However, all of the aforementioned 4D-CBCT prediction strategies neglected the spatiotemporal correlation inherent in 4D-CBCT.

To address the issues, we propose a combined model that contains 1) a convolutional LSTM (ConvLSTM) and 2) a principal component analysis (PCA) model with prior 4D-CT to map a single 2D measured projection to one phase of 4D-CBCT. We evaluated the model’s performance on both the XCAT phantom and pilot clinical data. Quantitative metrics are used for network performance quantification between our proposed method versus other state-of-the-art networks.

## 2 Methods

**Figure 1:**
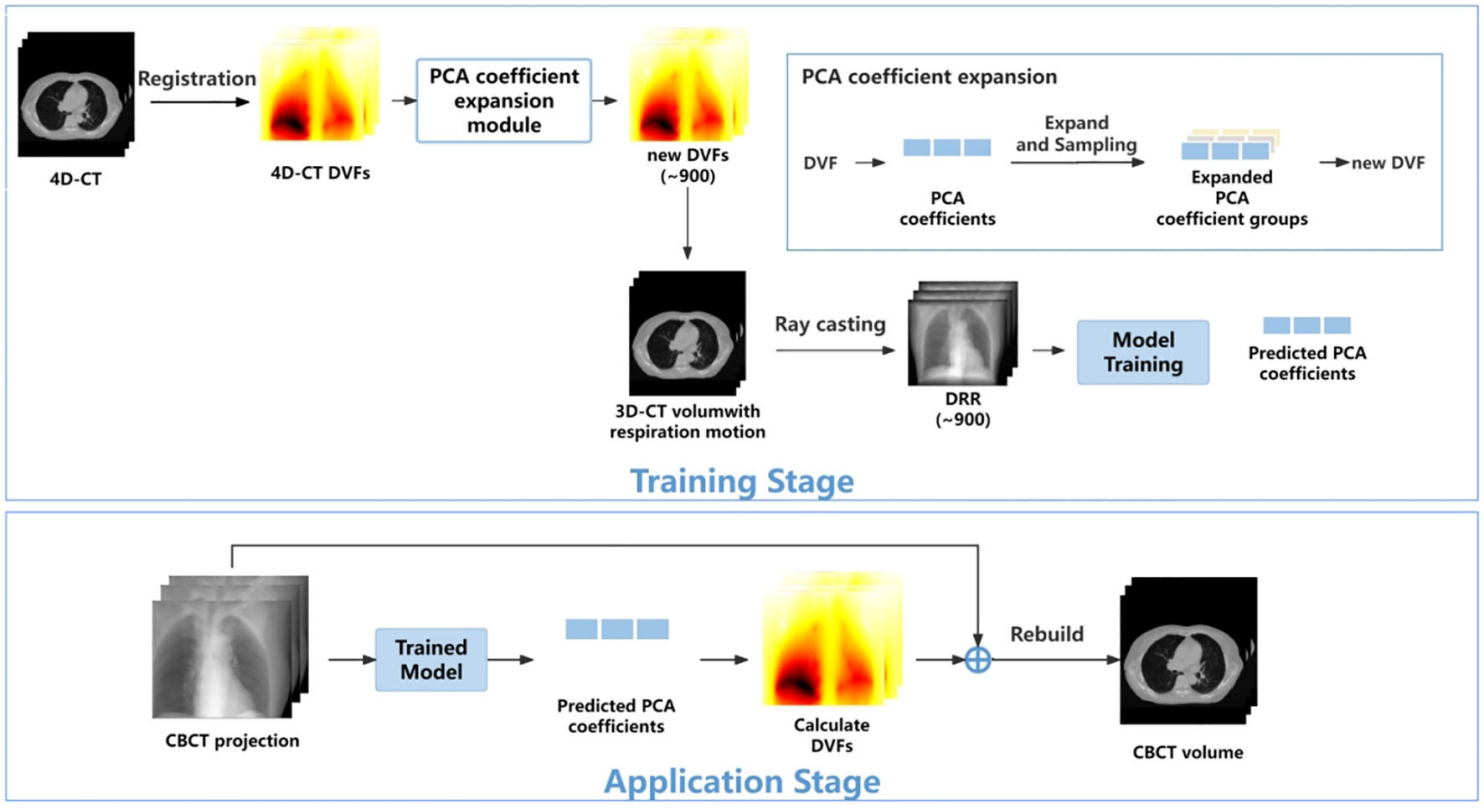
The workflow of the proposed method.

The overall workflow is illustrated in **Figure 1**. In the training stage, the 4D-DVFs are first derived from the 4D-CT (between 0% phase and other phases) via the voxel-by-voxel image registration algorithms (11–13). The DVFs then will be simply represented by the first few PCA coefficients. In our experiment, we chose the first three PCA coefficients. The PCA coefficient is further expanded to fully cover the potential possible motion range for simulation. We then performed random sampling and generated approximately 900 PCA coefficient groups. These groups will be used to create the corresponding 900 DVFs, which will in turn generate 900 deformed 4D-CT images with varying respiratory motions. Finally, a forward projection will be performed at a single angle for all 900 4D-CT images to acquire 900 DRRs. A ray-tracing algorithm (14, 15) is used in the forward projection simulation process. The generated DRRs will be used to train the ConvLSTM network, which has three output labels representing the first three PCA-modeled coefficients labels.

In the application stage, a single CBCT online projection that is measured at the same angle will be sent into the trained network. The network predicts three PCA labels to generate a phased 3D-CBCT. Then, more online projections (with different respiration amplitudes) will be continuously measured and sent into the network so that a whole respiration cycle will be covered. In this way, a full-cycle PCA label groups can be achieved and the whole 4D-CBCT. The entire process is performed on time. Below, we summarize our work into five parts: 1) motion modeling, 2) data processing, 3) network architecture, 4) loss function, and 5) experiment design.

### 2.1 Motion modeling

As mentioned above, the 4D-DVF is initially obtained from 4D-CT via deformable image registration (11–13). The 0% phase was selected as the reference phase to achieve the 4D-DVF. We used PCA, which is a commonly used data decoupling scheme for data dimension reduction (16), to extract DVF’s feature label (e.g., the principle components/eigenvectors). For computational efficiency consideration, we select the first three PCA labels for mapping the DVFs. **Table 1** illustrates the accuracy of DVF estimation relative to the number of PCA labels used. As expected, DVF accuracy improves with an increasing number of PCA labels. However, this also increases computational complexity. We found that by using the first three principal components, it already achieved 97.22% DVF information. Further increasing the PCA labels will not dramatically increase the information anymore. Therefore, we chose to discard the remaining PCA labels in our experiment.

**Table 1:** PCA label versus DVF estimation accuracy.

Number of PCA labels	information (%)	Increment of information (%)
1	71.08	71.02
2	87.37	16.35
3	97.22	9.85
4	98.24	1.02
5	99.20	0.96
6	99.62	0.42
7	99.89	0.27
8	100.00	0.11

The mapping relationship between the DVF and the PCA labels is given by Formula 1. Let the DVF size set be 3×*N_voxelCT_*, where *N_voxelCT_* stands for 3D-CT voxel number; 3 stands for the 3D motion. The DVF will be linearly mapped by Equation 1:


(1) $$DVF^{\left(i\right)}=\sum_{j=1}^{k}p_{j}^{\left(i\right)}q_{j}^{\left(i\right)}$$

Here, *p* and *q* stand for the eigenvectors and their corresponding PCA coefficients. Index *i* and *j* represent the respiration phase and eigenvectors, respectively.

### 2.2 Data processing

Being a regression task, ConvLSTM requires a large number of training data-set samples. In this study, we performed data augmentation and data enhancement. For data augmentation, we enlarged the simulated respiration amplitudes by a 15% interval up and down between two adjacent phases. This is because respiration is a time-continuous physiological motion. The concept of the 4D-CBCT phase is an average reconstruction for projections in one re-binned phase. The lung will move across the re-binned interface between two adjacent phases. Our extended motion amount covers just a bit more than the average motion range (7). This is to make sure all the possible motion amplitude will be modeled for training data generation. We perform PCA label random sampling to generate 900 DRRs as a training data set.

For data enhancement, we considered the influence of quantum noise in the simulated DRRs. Given that quantum noise is typically a combination of Poisson and Gaussian noise (17), we constructed a linear noise combination as follows see Equation 2:


(2) $$N=Poisson\left(I_{0}exp\left(-p_{n}\right)\right)+Gassian\left(0,\sigma_{e}^{2}\right)$$

$p_{n}$ is the noise-free signal line integral; the index N means the noise for each detector; $I_{0}$ is the X-ray projection intensity; and $\sigma_{e}^{2}\;$ represents background electronic noise. *I_0_* and $\sigma_{e}^{2}$ are set to be 10^5^ and 10, respectively. DRR was then added to the simulated noise to achieve the real projected image.

We also implemented an intensity correction scheme to minimize the intensity mismatch between the simulated training DRRs versus the measured CBCT projections. The correction is given by Equation 3:


(3) $$I_{DRR}^{\wedge }=\left(I_{DRR}-I_{Projection}\right)\times \frac{\sigma_{DRR}}{\sigma_{Projection}}+I_{DRR}$$

where $I_{DRR}^{\wedge }$ represents the corrected DRR intensity. $I_{DRR\;}$ and $\;\sigma_{DRR}\;$ represent the mean and the standard deviation of the original DRR intensity, and $I_{Projection}$ and $\sigma_{Projection}$ represent the mean and standard deviation of measured CBCT projection.

### 2.3 Network architecture

**Figure 2:**
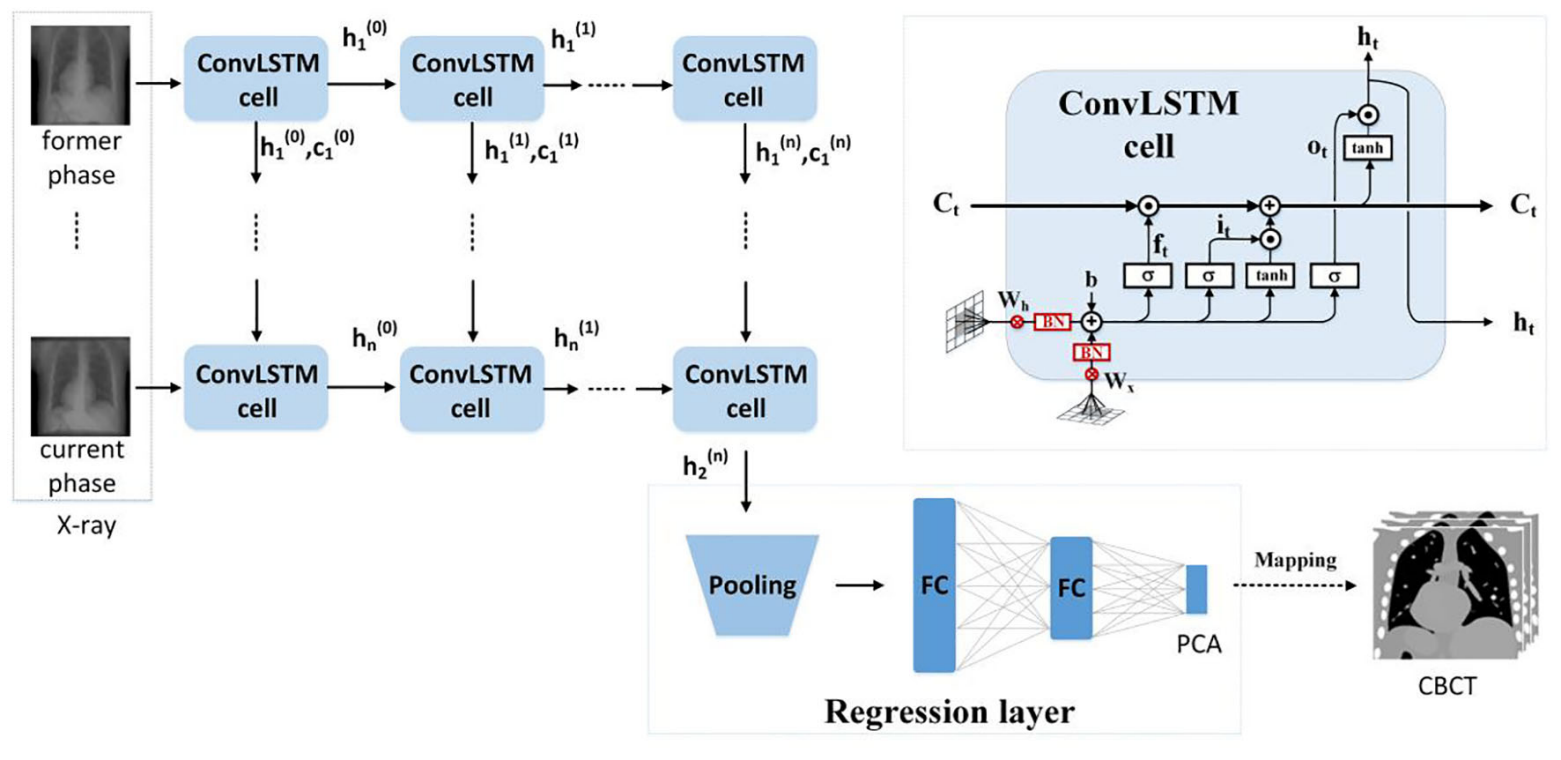
The ConvLSTM framework.

We use the ConvLSTM to explore the nonlinear mapping between DRRs and the PCA coefficients. The network architecture is illustrated in **Figure 2**. It contains a series of ConvLSTM cells and a regression layer.

Conventional LSTM (18) contains a memory cell ( $C_{t}$ ) and three gate control cells: 1) the forget grate ( $f_{t}$ ), 2) the input gate ( $i_{t}$ ), and 3) the output gate ( $o_{t}$ ). $C_{t}$ stores the foregone information, and the three gates update the cell. The LSTM sorts the relationships between all of the time flags; meanwhile, it ignores the internal information within each time flag. However, ConvLSTM (19), instead, explores the local features within each time flag via the convolutional operators. For the *t^th^* ConvLSTM cell, the internal operations will be represented by (19), see Equations 4–9:


(4) $$i_{t}=\sigma \left(W_{xi}*X_{t}+W_{hi}*H_{t-1}+b_{i}\right)$$

(5) $$f_{t}=\sigma \left(W_{xf}*X_{t}+W_{hf}*H_{t-1}+b_{f}\right)$$

(6) $$o_{t}=\sigma \left(W_{xo}*X_{t}+W_{ho}*H_{t-1}+b_{o}\right)$$

(7) $$G_{t}=tanh\left(W_{xg}*X_{t}+W_{hg}*H_{t-1}+b_{g}\right)$$

(8) $$C_{t}=f_{t}\circ C_{t-1}+i_{t}\circ G_{t}$$

(9) $$H_{t}=o_{t}\circ tanh(C_{t})$$

*σ* is the sigmoid function, tanh stands for the TanHyperbolic function, ∗ and ∘ represent the convolutional operator and Hadamard product, respectively. $X_{t}$ is the input of the current cell, and $G_{t}$ is a candidate storage unit for information transmission. In addition, *W* and *b* denote convolution kernels and the bias terms. *W* and *b* have obvious meanings. For instance, $W_{xo}$ is the input–output gate convolution kernel, while $b_{i}$ is the input gate bias, etc.

Due to the characteristic of the convolutional operator, ConvLSTM can acquire both temporal and spatial information simultaneously (19–22). Our ConvLSTM network contains 40 hidden layers and 20 cell layers. Moreover, it has eight layers, kernel size is 3, padding is set as “valid”, and the stride of the convolution kernel is 1.

The regression layer uses the feature map generated from ConvLSTM to predict PCA coefficients. It contains a pooling layer with two fully connected layers. By using the dominant local information, the pooling layer reduces the computation cost. The pooling was set to twice the down-sampling, and the dimensions of the two completely connected layers are 1,024 and 3.

### 2.4 Loss function

The normalized mean square error builds the loss function and is given in Formula 5. The PCA coefficients (e.g., output labels in the network) in the loss function (see Equation 10) ensured that the first coefficient has the highest estimation accuracy.


(10) $$Loss=\frac{1}{N}\sum_{i=1}^{N}\|w_{coeff}\circ \left(y_{i}-G\left(x_{i},W\right)\right){\|}_{2}$$

*N* is the training sample number; $\|{\|}_{2}$ represents the L_2_ norm, and *o* is the element-wise product. $G\left(x_{i},W\right)$ is the output of the regression model. $x_{i}$ is the *i* ^th^ training image, $y_{i}$ is the PCA coefficient, and W is the network parameters. $w_{coeff}$ is the PCA coefficients weight, which is set to be [ $\frac{2}{\sqrt{6}},\frac{1}{\sqrt{6}},\frac{1}{\sqrt{6}}$ ].

For model training, the ADAM optimizer was utilized with a dynamic learning rate, initially set at 0.001. The batch size was set to 8, and the training ran for 200 epochs. In an environment configured with Python 3.7 and an NVIDIA GeForce RTX 4080, training the data for 200 epochs took approximately 36 h.

### 2.5 Experiment design

For network performance evaluation, we use XCAT phantom and patient 4D-CT for the quantification. For testing, we simulated an on-board CBCT projection and then sent it into the pre-trained network to predict PCA coefficients. The quantification evaluation labels that we used are 1) the Mean Absolute Error (MAE), 2) the Normalized Cross Correlation (NCC), 3) the Multi-scale Structural Similarity(SSIM), 4) the Peak Signal-to-Noise Ratio (PSNR), 5) the Root Mean Squared Error (RMSE), and 6) the Absolute Percentage Error (MAPE). MAE is used to quantify the accuracy of regression models. *y* and $\hat{y}$ represent the label and the predicted value of the model, and *i* stands for the index of the regression model. We have in Equation 11:


(11) $$MAE=\frac{1}{m}\sum_{i=1}^{m}\left|{\hat{y}}^{\left(i\right)}-y^{\left(i\right)}\right|$$

In addition, NCC and SSIM (Multi-scale Structural Similarity Index Measure) are used to evaluate the quality of the reconstructed image. See Equations 12 and 13. *S* and *T* represent slice data with size of *H*×*W* of the original image and the reconstructed image, respectively. μ, δ , and $\delta^{2}$ represent the mean, covariance, and variance of the slice image, respectively.


(12) $$NCC=\frac{\sum_{k=1}^{C}\sum_{i=1}^{H}\sum_{j=1}^{W}\left|S\left(i,j\right)-\mu_{s}\right|\left|T\left(i,j\right)-\mu_{T}\right|}{C\sqrt{\sum_{i=1}^{H}\sum_{j=1}^{W}{\left(S\left(i,j\right)-\mu_{s}\right)}^{2}{\left(T\left(i,j\right)-\mu_{T}\right)}^{2}}}$$

(13) $$SSIM=\frac{\left(2\mu_{s}\mu_{T}+C_{1}\right)\left(2\delta_{ST}+C_{2}\right)}{\left(\mu_{s}^{2}+\mu_{T}^{2}+C_{1}\right)(\delta_{S}^{2}+\delta_{T}^{2}+C_{2})}=l\left(s,T\right)\cdot cs\left(s,T\right)$$

PSNR is defined based on MSE (Mean Squared Error). See Equations 14 and 15:


(14) $$MSE=\frac{1}{N}\sum_{j}\|S\left(j\right)-T\left(j\right){\|}^{2}$$

(15) $$PSNR=10*loɡ10\frac{MAX^{2}}{MSE}$$

N is the image pixel number. MAX is the maximum possible pixel value.

The definition of RMSE is given in Equation 16:


(16) $$RMSE=\sqrt{\frac{\sum_{i=1}^{M}\sum_{j=1}^{N}(S_{ij}-T_{ij}^{*}{)}^{2}}{H\times W}}$$

MAPE is the average ratio of the absolute difference between the predicted value and the true value to the true value. The definition of MAPE is given in Equation 17:


(17) $$MAPE=\frac{1}{n}\sum_{j}\left|\frac{S_{j}-T_{j}}{T_{j}}\right|$$

## 3 Results

### 3.1 Network parameter optimization

**Figure 3:**
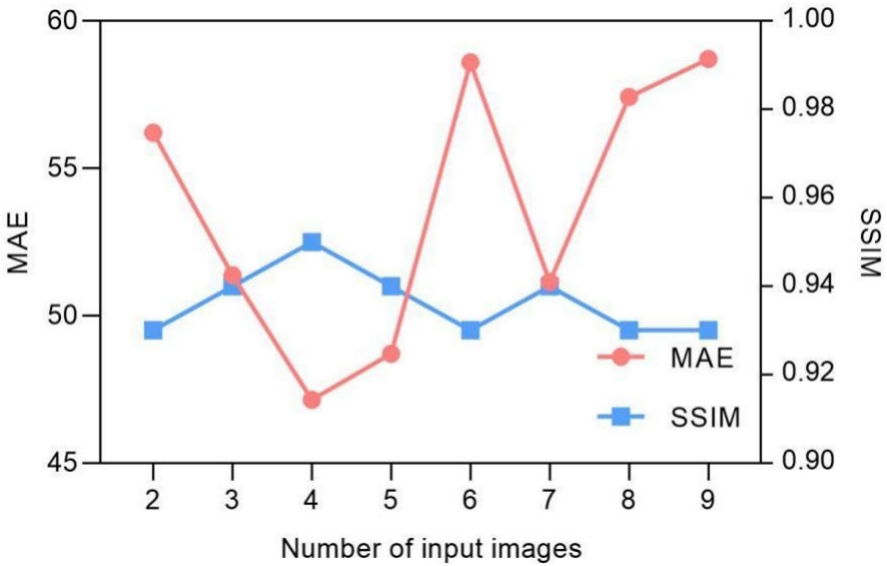
Input image quantity vs. MAE/SSIM of model prediction.

Being a spatiotemporal sensitive network, the temporal continuous image amount that the network can handle for data training reflects its ability for accurate motion estimation. However, **Figure 3** indicates that the model prediction accuracy is not dramatically influenced by the input image number. The MAE values fluctuate between 47 and 57, and the SSIM remains approximately 0.93. We found that the model achieves the best performance with four continuous temporal images with the lowest MAE of 47.15 and highest SSIM of 0.95.

**Figure 4:**
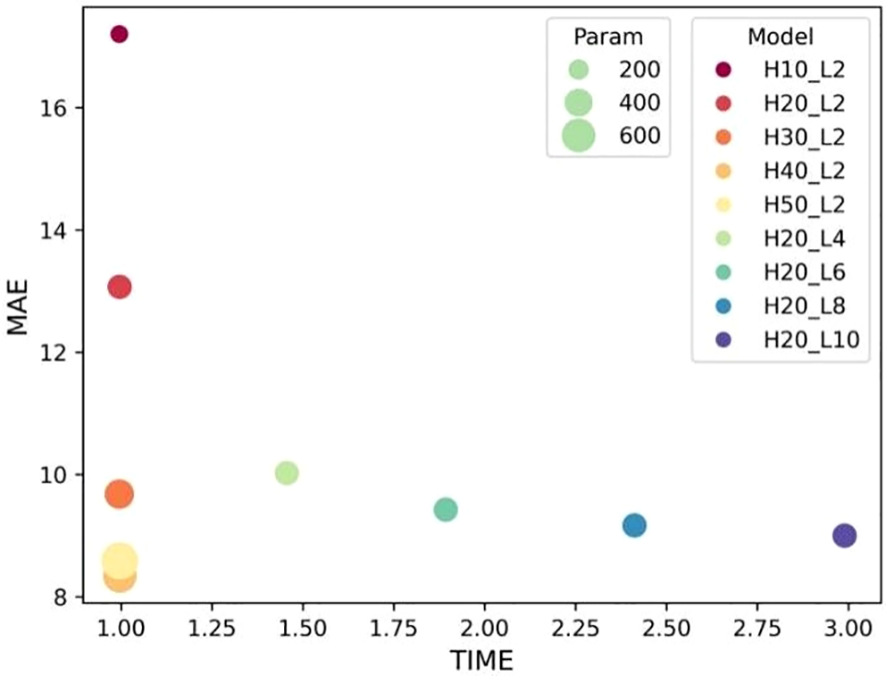
Influence of ConvLSTM cells on the model’s prediction. “H” stands for the number of hidden layers; “L” denotes the number of cell layers.

The selection of hyper-parameters for the ConvLSTM network was a critical aspect, as these parameters significantly impact the prediction performance of the model. To determine the optimal configuration, we conducted a series of ablation experiments focusing on the number of hidden layers and cell layers within the ConvLSTM network. The experiment results in **Figure 4** reveal that increasing the number of hidden layers decreased the MAE without significantly affecting computation time, although it did increase the number of parameters. Conversely, increasing the number of cell layers resulted in a slower decrease in MAE and an increase in computation time, with little change in parameter count. By balancing these factors, we determined that a configuration with 40 hidden layers and two cell layers provided the optimal trade-off, ensuring high prediction accuracy while maintaining computational efficiency.

### 3.2 Convergence of loss function

The convergence of the loss function is decided by the weightings. **Table 2** shows the convergence comparison caused by different weightings. Their MAE and NCC values are also summarized in the table. We found that the second group weighting (e.g., [ $2/\sqrt{6}$ , $1/\sqrt{6}$ , $1/\sqrt{6}$ ]) has the smallest first PCA label error. Meanwhile, this group also got the highest NCC.

**Table 2:** Weighting influence on MAE/NCC.

loss function weighting	MAE			NCC
	1st	2nd	3^rd^	
[ $1/\sqrt{3}$ , $1/\sqrt{3}$ , $1/\sqrt{3}$ ]	9.09	9.23	9.36	0.96
[ $2/\sqrt{6}$ , $1/\sqrt{6}$ , $1/\sqrt{6}$ ]	6.29	9.81	10.06	0.98
[ $\sqrt{3}$ / $\sqrt{6}$ , $\sqrt{2}$ / $\sqrt{6}$ , $1/\sqrt{6}$ ]	8.01	9.19	10.01	0.96

**Figure 5:**
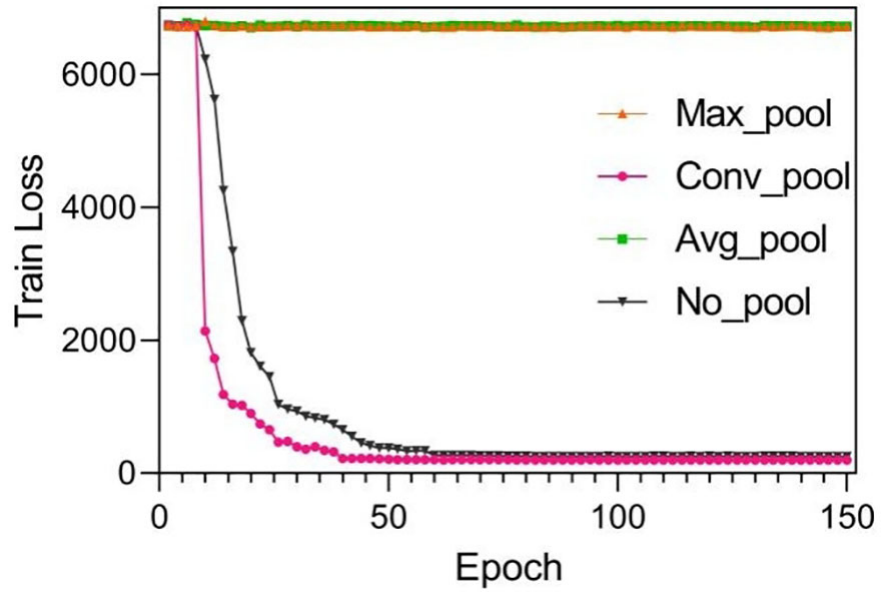
Training results by using different pooling optimizations. These pooling operations are set to twice the down-sampling, and the model only performs a single pooling operation.

Suitable choice of the pooling will also speed up loss function convergence. See **Figure 5**. The figure compared loss convergence curve with epoch with different pooling scheme such as Maximal pooling, Converlutional pooling, average pooling, and even no pooling at all. The results show that convolutional pooling achieves the best convergence performance. The pooling operation reduces the model’s parameters, hence accelerating its convergence.

**Figure 6:**
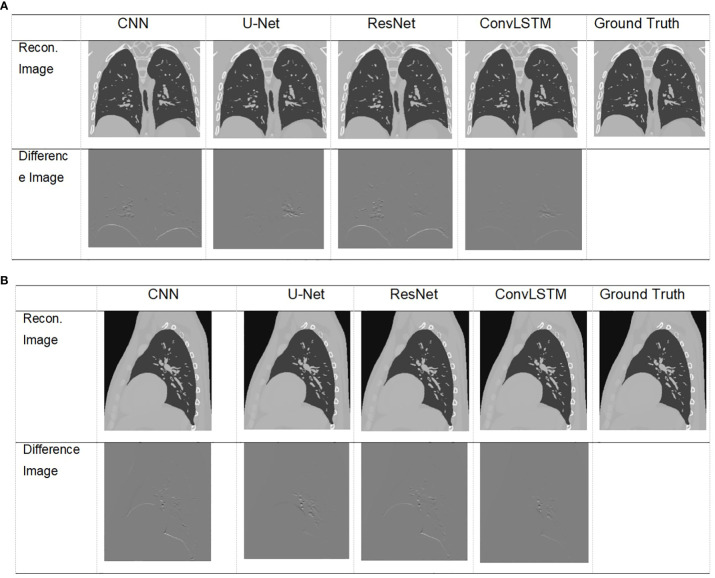
Visualization of images result of TestCase1 in different anatomical surfaces for each model with the training data generated from XCAT. **(A)** Coronal plane; **(B)** sagittal plane.

Suitable choice of pooling will also speed up loss function convergence. **Figure 6** compares the loss convergence curve with different pooling schemes such as maximal pooling, convolutional pooling, average pooling, and even no pooling. The results show that convolutional pooling achieves the best convergence performance. The pooling operation reduces the model’s parameters, hence accelerating its convergence.

### 3.3 XCAT simulation results

The XCAT phantom-based digital experiment was first performed. Four state-of-art network structures (e.g., CNN/Unet/ResNet/ConvLSTM) were tested with the phantom to compare their performances. As shown in **Table 3**, for the two test cases, the ConvLSTM outperforms other models in PCA coefficient prediction, especially for the first coefficient. The bold values provided in **Table 3** means that ConvLSTM achieves the best PCA coefficient match compared with that of the ground truth for XCAT phantom. By utilizing PCA to reduce the dimensionality of the DVFs, the ConvLSTM network focuses on the most significant components of respiratory motion. This not only improves computational efficiency but also ensures that the network is learning the most relevant features for accurate motion prediction. **Figure 6** presents the reconstructed results based on the PCA coefficients predicted by ConvLSTM versus CNN/UNet/ResNet. The reconstructed coronal plane and sagittal plane images and the different images between each reconstruction and the ground truth image are summarized in **Figures 6A, B**.

**Table 3:** Comparison of prediction results versus ground truth of XCAT data.

Model	PCA coefficients	
	Test Case1	Test Case2
CNN	[−1,121.3269 366.0489 −114.1392]	[5,736.9881 −391.8785 28.2141]
Unet	[−1,201.9354 394.0645 −66.5190]	[5,723.7266 −291.1034 56.5676]
ResNet	[−1,124.9048 378.9768 −60.8043]	[5,610.0141 −292.2644 90.5484]
ConvLSTM	[**−1,173.5433** 407.5900 −53.5265]	[**5,742.6875** −246.5791 181.1309]
Ground Truth	[**−1,163.5334** 454.6699 −78.2698]	[**5,787.5347** −258.0560 186.0697]

Values in bold indicate that our proposed method achieves the best quantification results compared to the ground truth.

**Table 4** summarizes the quantification evaluation comparison between each network. The results indicate that ConvLSTM outperformed other networks for all of the evaluation labels.

**Table 4:** Quantification comparison of prediction and reconstruction of each model on the coronal plane in XCAT TestData1.

Model	MAPE	PSNR	RMSE
CNN	0.2092	55.0287	0.0024
UNet	0.0464	62.0018	0.0015
ResNet	0.0628	56.6748	0.0025
ConvLSTM	0.0459	64.6742	0.0011

### 3.4 Pilot clinical results

**Figure 7:**
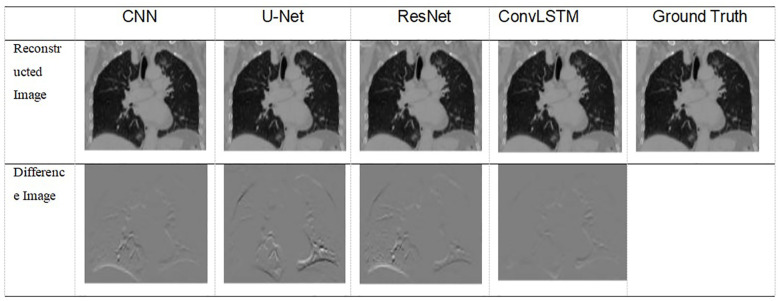
Visualization of images result of TestCase1 for each model with the training data generated from 4D-CT.

**Table 5** shows two cases of the real and predicted first three PCA coefficients of the patient data results. It is well known that the higher the principal component order, the higher the PCA contribution rate. As can be seen from **Table 5**, the first principal component of the model based on ConvLSTM is closest to the true value, just as the bold values illustrated. **Figure 7** shows the reconstructed coronal images based on the PCA coefficients predicted by CNN/UNet/ResNet and ConvLSTM network. We can see that all models have successfully reconstructed the anatomical structures, but ConvLSTM achieves the smallest different image to the ground truth. **Table 6** summarizes the quantification evaluation comparison between each network on the clinical TestCase1. According to the result, we can see that ConvLSTM supplies a prediction with the minimum error compared with the ground truth, certified that ConvLSTM outperformed other networks. Traditional CNNs and other networks mainly focus on spatial features, which limits their ability to accurately model dynamic processes like respiratory motion. The ConvLSTM’s ability to integrate convolutional operations with LSTM’s temporal processing allows it to effectively model the temporal evolution of respiratory motion, leading to more accurate 4D-CBCT reconstructions.

**Table 5:** Comparison of prediction results versus ground truth of patient data.

Model	PCA coefficients	
	Test Case1	Test Case2
CNN	[−676.5737 −36.4397 −26.6990]	[−87.5940 −117.7669 12.5394]
Unet	[−747.2873 −81.1768 −34.4381]	[−81.5389 −102.9164 11.1525]
ResNet	[−673.7071 −74.0461 −21.3157]	[−107.5652 −120.5355 14.5815]
ConvLSTM	[**−712.0823** −23.8481 −45.7298]	[**−99.6298** −112.0357 20.4654]
Ground Truth	[**−715.3792** −26.7257 −20.5198]	[**−101.5152** −127.8026 19.3956]

Values in bold indicate that our proposed method achieves the best quantification results compared to the ground truth.

**Table 6:** Quantification comparison of prediction and reconstruction of each model on the coronal plane in patient DataTest1.

Model	MAPE	PSNR	RMSE
CNN	0.2206	57.8427	0.0037
UNet	0.3313	53.6098	0.0060
ResNet	0.2706	55.4795	0.0048
ConvLSTM	0.0934	63.7294	0.0019

## 4 Discussion

In this study, we proposed a spatiotemporal consistent scheme via ConvLSTM and PCA motion modeling to estimate online 4D-CBCT. The network learns the motion features from patient 4D-CT with hundreds of simulated DRRs under a fixed angle. Both digital XCAT phantom experiments and pilot clinical studies were performed to prove the algorithm’s efficiency. We compared our proposed method’s efficiency with other popular networks such as CNN/Unet/ResNet. Quantification results indicate that ConvLSTM outperforms its competitors. ConvLSTM is an architecture that integrates Convolutional Neural Networks (CNN) with Long Short-Term Memory (LSTM) networks, enabling the application of convolution operations at each time step to effectively capture spatial information in temporal data. Compared to CNN, U-Net, and ResNet architectures, ConvLSTM can link the feature information of the current projection with that of adjacent projections, providing enhanced temporal and spatial feature connectivity. Hence, it will be able to supply enough information for motion estimation with temporal correlation.

In this work, our goal is to develop a real-time 4D-CBCT imaging model utilizing projection images with high temporal resolution. The model inference for PCA labels is remarkably fast, taking approximately 0.006 s for one projection. This rapid inference is critical for maintaining real-time processing capabilities, ensuring that the model can handle a continuous stream of projection images without significant latency. However, the reconstruction time for a single volume of 4D-CBCT is approximately 5 s on a personal desktop computer. While this is relatively fast given the complexity of the task, it underscores the computational demands associated with high-resolution 4D imaging. Our ongoing work focuses on optimizing this reconstruction time further, possibly through hardware acceleration or more efficient algorithms, to achieve even faster performance.

Despite the promising results, our study has several limitations that need to be addressed. First, the study relies on simulated data for training the network, including simulated respiratory motion and noise models. While these simulations aim to mimic real-world conditions, they may not fully capture the complexities of actual patient data, potentially affecting the model’s performance in clinical settings. Second, the proposed model depends heavily on the consistency of the patient’s respiration pattern between the initial 4D-CT scanning and the online treatment stages. Any significant variation in the patient’s breathing pattern during treatment could impact the accuracy of the 4D-CBCT reconstruction. Third, the pilot clinical evaluation was conducted with a limited number of patients. Although the results were promising, a larger and more diverse patient cohort is necessary to validate the robustness of the proposed method.

## Data availability statement

The original contributions presented in the study are included in the article/supplementary material. Further inquiries can be directed to the corresponding authors.

## Ethics statement

The studies involving humans were approved by Affiliated Hospital of Jiangsu University Review Board (#2016-034). The studies were conducted in accordance with the local legislation and institutional requirements. Written informed consent for participation in this study was provided by the participants’ legal guardians/next of kin.

## Author contributions

HZ: Writing – original draft, Writing – review & editing, Methodology, Supervision. KC: Investigation, Software, Writing – original draft. XX: Data curation, Software, Writing – original draft. TY: Validation, Writing – review & editing. WS: Funding acquisition, Writing – review & editing. JD: Writing – original draft, Writing – review & editing.
